# 10-Year Locoregional Control with Postoperative External Beam Radiotherapy in Patients with Locally Advanced High-Risk Non-Anaplastic Thyroid Carcinoma De Novo or at Relapse, a Propensity Score Analysis

**DOI:** 10.3390/cancers11060849

**Published:** 2019-06-19

**Authors:** Stéphanie Servagi Vernat, Jonathan Khalifa, Xu-Shan Sun, Emmanuel Kammerer, Eivind Blais, Jean-Christophe Faivre, Terence Tai-Weng Sio, Jianji Pan, Hao Qiu, Gil Bar-Sela, Jean-Marc Simon, Julia Salleron, Juliette Thariat

**Affiliations:** 1 Department of Radiation Oncology, Institut Godinot, 51100 Reims, France; 2 Department of Radiation Oncology, Institut Universitaire du Cancer, 31100 Toulouse, France; jonathan.khalifa@hotmail.fr; 3 Department of Radiation Oncology CHU, Besançon-Montbeliard, 25200 Montbeliard, France; xssun@chbm.fr; 4 Baclesse Cancer Center/ARCHADE, 14000 Caen, France; emmanuel.kammerer@gmail.com (E.K.); jthariat@hotmail.com (J.T.); 5 Unicaen-Normandie University, 14000 Caen, France; 6 Laboratoire Physics Lab, 14000 Caen, France; 7 Department of Radiation Oncology, Hospital Pitie Salpetriere, 75013 Paris, France; eivind.blais@gmail.com (E.B.); jean-marc.simon@aphp.fr (J.-M.S.); 8 Department of Radiation Oncology, Lorraine Institute of Cancerology, 54519 Nancy, France; jc.faivre@nancy.unicancer.fr; 9 Department of Radiation Oncology, Mayo Clinic, Phoenix, AZ 85054, USA; sio.terence@mayo.edu; 10 Department of Radiation Oncology, Fujian Provincial Cancer Hospital, Fuzhou 350014, China; panjianji@126.com; 11 Department of Radiation Oncology, Cancerology Center, 41260 La Chaussee Saint Victor, France; qiuhao2007@gmail.com; 12 Department of Radiation Oncology, Rambam Health Care Campus, 31096 Haifa, Israel; gilbarsela1@gmail.com; 13 Biostatistics, Lorraine Institute of Cancerology, 54519 Vandœuvre-les-Nancy, France; j.salleron@nancy.unicancer.fr

**Keywords:** thyroid carcinoma, non-anaplastic, radiotherapy, surgery, locoregional failure

## Abstract

(1) Background: To assess the role of postoperative external beam radiotherapy (pEBRT) on locoregional failure (LRF) for patients with locally advanced high-risk non-anaplastic thyroid carcinoma (naTC) at primary event or relapse. (2) Methods: Between 1995 and 2015, postoperative naTC patients with a theoretical indication for EBRT were included based on criteria that were common to American-British-French current guidelines, i.e., pT3-4, pN+, gross or microscopic residual disease. Inverse probability of treatment weighting (IPTW) after multiple imputation was used to reduce selection biases. (3) Results: Of 254 naTC patients, 216 patients underwent pEBRT (106 de novo, 110 at relapse, median dose 60 Gy) and 38 underwent surgery only. pEBRT patients had more gross residual disease, a major prognostic factor (*p* = 0.027) but less perineural invasion (*p* = 0.008) or lymphovascular emboli (*p* = 0.009). pEBRT patients more frequently underwent radioiodine therapy (*p* = 0.026). The 10-year cumulative incidence of LRF was 56% (95% CI, 32–74%) in operated patients, and 23% (95% CI, 17–30%) in pEBRT patients. After IPTW method, pEBRT reduced the risk of LRF (hazard ratio 0.30; 95% CI [0.18–0.49], *p* < 0.001), but had no impact on OS. In the pEBRT group, non-Intensity Modulated RadioTherapy (IMRT) plans and interruption of the radiotherapy were associated with poorer survival, while extended versus limited field strategy and dose were not. (4) Conclusions: In naTC patients who have pT3-4, pN+ disease or R1-2 resection, pEBRT improved LRF. Limited-field IMRT is preferred.

## 1. Introduction

Thyroid cancer represented 53,000 new cases in 2012 (2% of all cancers) in Europe, and is increasing in incidence [1]. Most thyroid cancers originate from glandular epithelial cells and are sub-divided into papillary, follicular, poorly differentiated thyroid carcinoma (PDTC), medullary and anaplastic thyroid carcinomas, with prevalences of 80%, 11%, 4%, <1% and 2%, respectively [2]. The mainstay of treatment of non-anaplastic thyroid carcinomas (naTC) is surgery (lobectomy or total thyroidectomy, with or without neck dissection). It is often followed by adjuvant radioactive iodine therapy (RAI) [3,4]. Locoregional recurrence occurs in 15% of patients with naTC, despite RAI and more patients becoming RAI refractory at relapse [5]. Postoperative external beam radiotherapy (pEBRT) has been used to improve locoregional control; however, with conflicting results in the literature and inconsistent guidelines across countries [3,6]. The only trial evaluating the clinical benefit of EBRT for locally advanced naTC was closed due to poor accrual, and differences in recurrence rates between the irradiated and control arms in the extended observational study were not significant [7]. Considering that pEBRT may only be beneficial in a small subset of patients, we evaluated whether pEBRT improved locoregional control in the management of operated patients with locally advanced high risk (with poor prognostic factors: pT3-4, pN+ or R1-2) naTC after surgery de novo or at relapse, compared to patients undergoing surgery only.

## 2. Results

### 2.1. Patient and Treatment Characteristics

Of 254 patients from 18 radiation therapy departments, 216 patients underwent pEBRT (85.0%) and 38 had surgery without EBRT (15.0%; control group). Patient and tumor characteristics are presented in Table 1. Twenty-four (10.7%) T0-Tx patients were referred for management of nodal disease.

Thirty-eight (16.9%), 15 (6.8%) and 6 (2.7%) patients had tracheal, esophageal and laryngeal invasion, respectively. One hundred and seventy-one (68.1%), 22 (8.9%), 31 (12.5%) and 38 (15.3%) patients had papillary, follicular, PDTC, and medullary carcinomas, respectively.

Total thyroidectomy was performed in 217 (86.5%) patients, partial thyroidectomy or debulking surgery was used in the others, and 209 had a neck dissection (86.4%). Sixty-seven (30.5%) and 40 (18.2%) patients had microscopic or gross residual disease, respectively. pEBRT patients had more microscopic and macroscopic residual disease (R1, 33.2% vs. 15.1%, R2, 19.2% vs. 12.1%, *p* = 0.027). pEBRT patients had significantly less perineural invasion (14.2% vs. 40.0%, *p* = 0.008), vascular emboli (28.3% vs. 51.7%, *p* = 0.009) and lymphatic emboli (18.7% vs. 42.3%, *p* = 0.009). Fifty-eight patients (24.0%) underwent RAI, more frequently in pEBRT patients (26.4% vs. 8.8%, *p* = 0.026). Overall, 35 patients (13.8%) had chemotherapy; 32 pEBRT patients and 3 in the surgery group. In pEBRT patients, one and 10 had preoperative or postoperative chemotherapy, respectively and 21 patients had concomitant chemoradiotherapy with weekly adriamycin or carboplatin. 

Among pEBRT patients, 106 were irradiated at primary event and 110 at relapse. A limited field technique (with irradiation of high-risk volume only) was used in 30 (14.1%) and an extensive field technique (including operative bed, thyroid area and lymph node areas prophylactically) in 183 (85.9%). IMRT or 2D/3D radiotherapy was used in 74 (36.8%) and 127 (63.2%) patients, respectively. Median dose to the high-risk volume (macroscopic disease) and intermediate/low-risk volumes (microscopic disease and prophylactic volume) was 60 Gray (Gy) (interquartile range (IQR), 56 to 66) and 50 Gy (IQR, 45 to 54), respectively. Thirty patients (13.9%) had > 66 Gy. Daily 2-Gy fractions were used in 103 (89.4%) patients.

### 2.2. Outcomes

Median follow-up was 78 months (range, 41–122 months). Sixty-eight patients (26.8%) had LRF: 15 both local and nodal failure, 15 local failure only, and 32 nodal failure only (unspecified site in 6). Of 46 pEBRT patients, 17 relapsed in the high-risk volume, 6 in the intermediate/low-risk volume, 2 had a marginal relapse, and 1 an out-of-field relapse (dose pattern of relapse not addressed in 20 patients).

Two-, 5- and 10-year cumulative incidences of LRF were 8.6% (95% CI; 5.5–12.5%), 18.6% (13.8–23.7%) and 28.1% (2.1–34.3%), respectively. Ninety-one patients had metastases at the time of analysis; 10-year incidence was 34.8% (28.5–41.3%). Two, 5- and 10-year overall survival rates were 88.6% (95% CI; 83.9–92.0%), 78.4% (72.6–83.2%) and 63.0% (55.4–69.6%) respectively.

### 2.3. Impact of pEBRT

**Figure 1:**
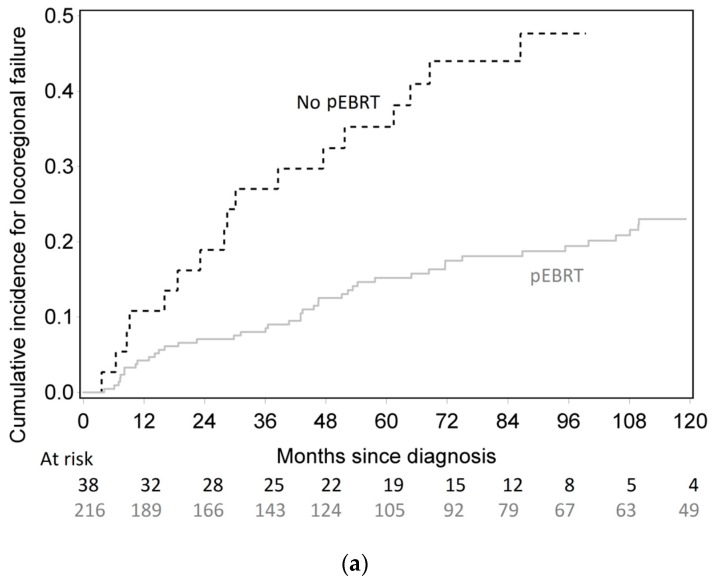

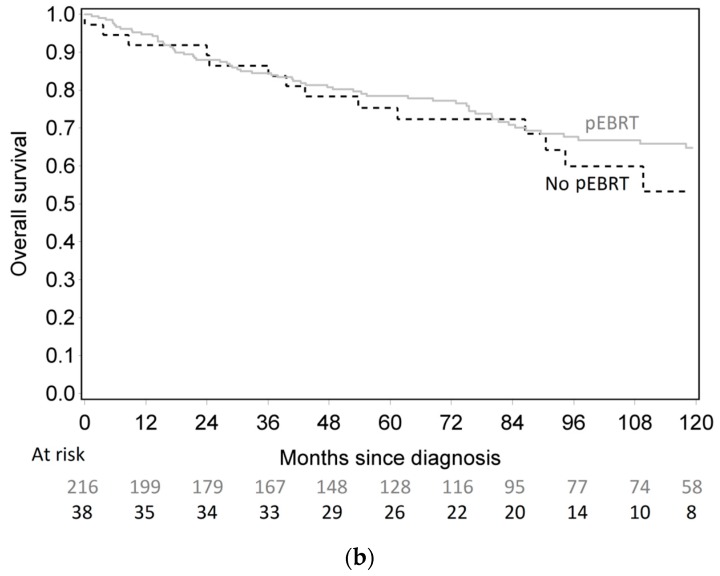
(**a**) Cumulative incidence of locoregional failure according to postoperative external beam radiotherapy (pEBRT) and surgery group. (**b**) Overall survival according to pEBRT and surgery group.

**Figure 2:**
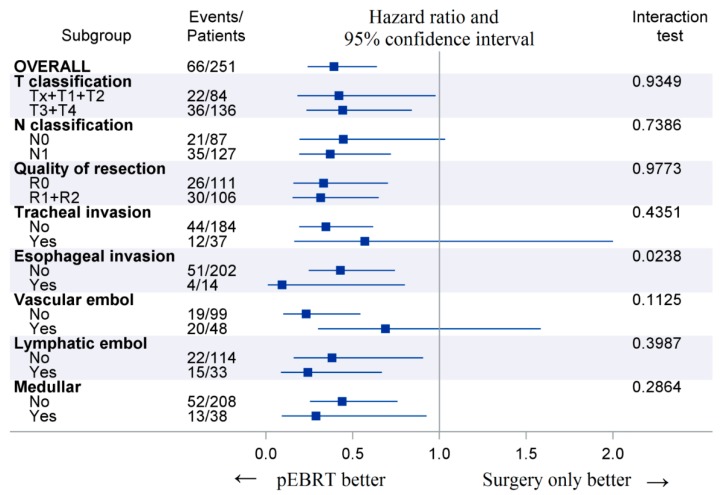
Forest plots displaying postoperative external beam radiotherapy (pEBRT) effects on locoregional failure across subgroups. Legend: Events represent the number of locoregional failures at last follow-up. The *p*-value is from the test statistic for testing the interaction between pEBRT and any subgroup parameter.

The 10-year cumulative incidence of locoregional recurrence was significantly lower in pEBRT patients, with 23% (17–30%) compared to 56% (32–74%) for patients without pEBRT (HR 0.30; 95% CI (0.18–0.49), *p* < 0.001) (Figure 1). This impact was similar in subgroup analyses as there was no interaction between pEBRT and patient/tumor characteristics (Figure 2) except for patients with esophageal invasion for whom the benefit of pEBRT was higher. After multiple imputation and inverse probability of treatment weighting (IPTW) method, pEBRT still reduced the risk of LRF (HR 0.17; 95% CI, 0.10–0.29, *p* < 0.001). Similarly, in the sub-group of medullar thyroid cancer patients, LRF remained lower in pEBRT patients (HR 0.25; 95% CI, 0.13–0.49, *p* < 0.001). pEBRT had no significant impact on overall survival (0.82, 95% CI, 0.41–1.64, *p* = 0.600) (Figure 1b). 

### 2.4. Acute and Late Toxicities of Radiotherapy

In 24 patients (11.1%, 9 patients for toxicity), radiotherapy was interrupted for over three days. There was no difference between 2D/3D and Intensity Modulated RadioTherapy (IMRT) (5.5% vs. 2.7%, *p* = 0.49). Acute G3-4 mucositis occurred in 8 patients (3.7%), dysphagia in 15 (6.9%), dysphonia in 8 (3.7%), dermatitis in 1 (0.5%), and aspiration in 5 (2.3%). One patient had late G3 radiation pneumonitis, 1 had a maxillary fracture, 1 had fibrosis, 1 had severe xerostomia and 5 (2.3%) had spinal nerve deficit. Rates of acute and late G3-4 toxicities were similar between 2D/3D and IMRT (*p* = 0.63 and 0.50, respectively).

### 2.5. Prognostic Factors in pEBRT Patients 

In multivariate analysis (*n* = 216), total thyroidectomy was associated with a lower risk of LRF, while poorly differentiated thyroid carcinoma was associated with increased risk of LRF (Table 2). Interruption of radiotherapy for over three days increased the cumulative incidence of LRF in univariate analysis. Table 3 shows the prognostic factors of overall survival in the pEBRT group. Radioiodine treatment improved overall survival, while age >45, laryngeal invasion, 2D/3D radiotherapy (versus IMRT) and interruption of radiotherapy were associated with an increased risk of death. 

## 3. Discussion

Using FDA-approved multiple imputation methods, we were able to adjust for known selection biases and differences in pre-treatment and baseline characteristics by using IPTW method. Results between complete cases analyses and results after MI were of course not strictly identical but fully consistent, thus increasing confidence in the conclusions reached and in the robustness of the analyses. Doing so, we showed that pEBRT improved LRF after surgery for de novo or relapsed naTC patients with pT3-4 or pN+ disease or incomplete resection margins, following American-British-French guidelines, and allowing RAI and any age. pEBRT reduced the risk of LRF with a hazard ratio of 0.17 in patients. Other prognostic factors, such as perineural invasion, vascular emboli, lymphatic emboli, which are unusual prognostic factors in thyroid naTC, were less likely present in irradiated patients and vascular emboli were associated with poorer LRF. In contrast, major unfavorable factors such as R1-2 resection was more frequent in pEBRT patients. Additionally, patients undergoing surgery alone more frequently had de novo disease compared to pEBRT patients, of whom half had recurrent disease. It is not surprising that radiotherapy was more frequently prescribed in locoregional naTC at relapse. The guidelines recommend the use of postoperative pEBRT in recurrent tumours that fail to concentrate radioactive iodine and for which additional surgery would be ineffective [3,4,6,8]. Intriguingly, despite reluctance of French surgeons to advocate radiotherapy in naTC, pEBRT patients overall more frequently underwent RAI than in surgery alone patients. RAI did not improve LRF and adjustment performed on RAI confirmed that the benefit of pEBRT was not biased by RAI. Consistently, Tam et al. recently showed a benefit of pEBRT in patients receiving RAI [9]. As for perineural invasion and lymphovascular emboli, these criteria have been little used in naTC, because they are not identified as strong prognostic factors of relapse. They were less frequently present in pEBRT patients. Whether this selection bias explains the benefit of pEBRT in patients with any other well-known major prognostic factor is unlikely but cannot be excluded. Being considered minor factors, the above-mentioned criteria were inconsistently described on operative reports. We used FDA-approved multiple imputation methods to have an unbiased estimate of these factors on all patients despite a significant number of missing data. Lymphatic emboli and perineural invasion did not influence the risk of LRF in pEBRT patients. In contrast, vascular emboli were an independent risk factor of LRF [10]. In the future, pathologists should be encouraged to report on the presence of perineural invasion, lymphatic and vascular emboli systematically.

One additional confounding factor could have been chemotherapy. However, chemotherapy did not influence LRF or survival in our series and thus did not contribute to the benefit observed with EBRT. It is important to investigate further because some series report on high rates of prescription of chemotherapy [11,12]. 

Finally, a benefit of EBRT has been shown in several retrospective studies [13,14,15,16,17]. Our practical approach was to investigate the role of pEBRT in patients that are referred for pEBRT based on poor prognosis criteria that are common to American-British-French guidelines. pEBRT seems to provide a benefit on locoregional control under the condition that at least a so-defined criterion (pT3-4, pN+, R1-2) is present. For this very reason, and in contrast to other series, it is thus not surprising that our series has “inverse proportions” of pEBRT versus surgery only. In Chow’s study of 842 operated patients including 105 irradiated patients, pEBRT reduced the risk of LRF to 0.35. Our current study similarly demonstrated a risk of decreased incidence of LRF with a hazard ratio of 0.17. We confirmed that pEBRT improved locoregional control after addressing the selection biases by using IPTW method. Additional poor prognostic factors, such as age, laryngeal invasion, non-IMRT and radiotherapy interruption, further influenced the magnitude of the benefit.

However, there was no benefit of pEBRT in our series with respect to overall survival. This might be explained by the fact that we only assessed overall survival and not naTC-specific deaths. Because naTC patients have relatively long life expectancies, deaths from non-cancer causes may also occur. In Brierley’s study, cause-specific survival was 81% and 64.4% (*p* = 0.04) with EBRT and no EBRT, in patients >60 years of age with microscopic residual disease and no gross residual disease [8]. Chow et al. demonstrated improved 10-year cause-specific survival from 49.7% to 74.1% (*p* = 0.01) with EBRT in patients with gross residual disease [13], leaving the question open with respect to impact of pEBRT on survival in select populations. Moreover, in high-risk patients, relapse after treatment can also be associated with increased rates of metastatic-related deaths, which are competitive events for locoregional progression free-survival.

One puzzling observation was that conformal (non-IMRT) radiotherapy was associated with poorer survival compared to IMRT but we could neither show an impact of the technique on locoregional failure nor on toxicities. We showed low frequencies of high-grade toxicities overall. Schwartz et al. reported a lower complication rate with IMRT (12% with 3D irradiation and 2% with IMRT) [18]. Overall, IMRT lowers the frequency and severity of toxicities (such as late dysphagia) and should be performed in all naTC patients. Reporting on severe toxicities only in our series was intentional, because it is often difficult to determine whether mild-moderate toxicities were not reported, because they did not occur or because they were not collected. Treatment interruption was identified as a new poor prognostic factor in naTC. Another important observation was that limited field EBRT did as well as extended EBRT with prophylactic volumes, suggesting that irradiation may be limited to gross tumors with reduced margins rather than systematic irradiation of the whole neck and anterior mediastinum. Numbers were, however, small. A reason for better overall survival after IMRT vs. 3DCRT may be a correlation with a general time trend towards increasing overall survival in cancer survivors and also in the healthy population.

Limitations of our study include small patient numbers in the surgical group. The most likely explanation is that selection was based on limited selected poor prognosis criteria for pEBRT based on American-British-French current guidelines. Another limitation is that we included all non-anaplastic thyroid carcinomas including medullary and poorly differentiated thyroid carcinomas. Similarly, in the sub-group of patients without medullar thyroid cancer patients, LRF remained lower in pEBRT patients after IPTW method (HR 0.25; 95% CI, 0.13–0.49, *p* < 0.001). However, patients with medullary thyroid cancer did not receive radioiodine per standard of care and also benefited from pEBRT; thus, the question of pEBRT is also relevant in this subtype of non-anaplastic carcinomas [19]. Similarly, PDTC had poorer prognosis, but also benefitted from pEBRT. 

## 4. Material and Methods

### 4.1. Study Design

This Institutional Review Board- and ethical committee-approved was retrospective and multicentric study. It included consecutive naTC patients that had a theoretical indication for pEBRT based on poor prognostic factors according to current American-British-French guidelines, i.e., pT3-4, pN+ or R1-2. Patients were included regardless of radioiodine uptake. All patients underwent primary or salvage surgery of their thyroid with or without cervical neck dissection between November 1995 and 2015. Stage was determined using the seventh edition of the American Joint Committee on Cancer staging system. In addition to inclusion, data collection included patient-related criteria, additional histological criteria and therapeutic parameters. Patient-related data included age, gender, and performance status. Cancer-related data included staging (primary and nodal stage), tracheal/esophageal/laryngeal invasion, lymphatic, vascular emboli and perineural invasion, and number of involved nodes, and radioiodine uptake. Treatment-related data included (1) surgery (extent of thyroid resection, lymph node dissection, quality of resection, extrathyroidal extension, microvascular invasion, perineural invasion, vascular emboli, and/or lymphatic emboli); (2) Radioiodine treatment (RAI); (3) radiotherapy technique (Conformal (2D/3D) and intensity modulated (IMRT) radiation therapy), total dose, number of fractions, target volumes (limited field (tumor bed only), or extensive field (tumor bed and prophylactic thyroid and lymph node areas)), interruption and duration of radiotherapy; (4) Chemotherapy (concomitant, neoadjuvant, adjuvant, type). 

### 4.2. Follow-Up

Patients underwent follow-up visits according to institutional standards, usually every 3 months for the first 2 post-operative years, then every 6 months for up to 5 years, and yearly thereafter. Follow-up evaluations included a physical examination, and complete blood count, liver function test, serum thyroglobulin level, neck US and/or CT, 131I whole body scan if necessary. Acute and late toxicities were collected according to Common Terminology Criteria for Adverse Events (CTCAE v4.0). 

### 4.3. Statistical Methods

Qualitative parameters were described as frequencies and percentages, and continuous parameters as mean and standard deviation or by median and inter-quartile range according to the normality of the distribution as assessed by the Kolmogorov test. Patient and treatment characteristics were compared according to the presence or absence of pEBRT with Chi-square or Fisher Exact test. Patients after surgery alone or after radiotherapy (definitive or Post-operative) underwent follow up visits every 3 months for the first 2 post treatment years, then every 6 months for up 5 years. LRF status (relapse or progression) was assessed at each time point of follow up. We considered the recurrence to be a LRF when a newly suspicious lesion was detected in operated patients (+/− radiotherapy) on follow up CT and/or Iodine-131 whole-body scintigraphy. Needle aspiration cytology of any suspicious lesion was performed to confirm failure and imaging was repeated 3 months later in inconclusive cases. In patients with gross residual disease: 

An increase in the size of the lesion on CT compared with pretherapeutic CT was considered as LRF (>20% according to the RECIST criteria) during the evaluation (3,6,9 months). When Iodine-131 whole-body scintigraphy was performed, LRF could also be defined by the presence of a significant hypermetabolism on Iodine scintigraphy.

Progression could also be detected on Positron Emission Tomography-Computed Tomography (PET-CT) in case of refractory disease (deemed to be iodine-negative). LRF was described with cumulative incidence methods while considering metastatic recurrence and death as competing events [20]. The impact of patient, tumor and initial treatment characteristics on LRF was evaluated using the Fine and Gray test [21]. Exploratory subgroup analyses were performed to investigate the interaction between pEBRT and any subgroup characteristics and were illustrated by forest plot. Overall survival (OS) was determined by the Kaplan-Meier method and prognostic factors were tested using the Cox proportional hazard model. All time points were computed since date of diagnosis. 

The impact of pEBRT on LRF and OS was first assessed in bivariate analyses. To adjust the results on selection biases (i.e., the choice of pEBRT could be done according to tumor and patient characteristics), the results of these bivariate analyses had to be adjusted on major prognostic factors. The IPTW method was applied for the adjustment [22]. Due to missing values, multiple imputations (MI) on patient, tumor and initial treatment characteristics were performed according to FDA-approved methods [23]. Since this study was retrospective in nature, we assumed that the described observations were missing at random (MAR) [24]. Multiple pilot runs of various numbers of imputations were performed to assess the number of imputations and the stability of the parameter estimates for a given number of imputations. The number of 40 imputations was defined according to the fraction of missing information and the relative efficiency [25]. The following method was applied: (a) multiple imputation using fully conditional specification was performed on 40 datasets using the proc mi in Statistical Analysis System (SAS), version 9.4; the multiple imputation model was performed on all described patient, tumor and initial treatment characteristics. The survival time and the time to locoregional failure were also included. (b) For each imputed dataset, the propensity score was computed with either the presence or absence of pEBRT as dependent parameters, and with all described patient, tumor and initial treatment characteristics and the inverse probability of treatment—pEBRT—was computed [10]. (c) The effect of pEBRT on LRF was estimated by the hazard ratio using a survival model using the IPTW method [22]. (d) The 40 hazard ratios were then combined across imputed datasets with the proc mi analysis. A sensitivity analysis was also performed on the sub-group of patients without medullary thyroid cancer.

In pEBRT patients, prognostic factors of LRF and OS survival were evaluated after multiple imputations to reduce bias and to increase the precision of the estimates [26]. First, each prognostic factor was tested by bivariate analysis on each imputed dataset and hazard ratios were then combined across imputed datasets with the proc mi analysis. Results of the bivariate analyses were presented as hazard ratio and 95% confidence intervals. Parameters with a *p*-value less than 0.1 in bivariate analysis were selected for the multivariate analysis. To simplify the full model, a backward selection was performed in each imputed data set resulting in models with different selected predictors. Selected predictors for the optimal model were predictors appearing in at least half of the models [27]. The optimal model was then computed with the selected predictors in each imputed data set and models were then combined across imputed datasets with the proc mi analysis. Results of the optimal multivariate model were presented as adjusted hazard ratio and 95% confidence interval. Based on FDA recommendations, the number of individuals with missing data for each variable of interest, as well as the results of the complete-case analysis (such as sensitivity analysis), are presented in the supplementary sections (Tables S1–S3) to evaluate the confidence in the conclusions reached and in the robustness of the analyses [26,28]. For the analysis of prognostic factors of LRF and OS on complete cases, each prognostic factor was tested in bivariate analyses and the results are presented as a hazard ratio and 95% confidence interval. The parameters with a *p*-value less than 0.1 in bivariate analysis were selected for the multivariate analysis. To simplify the full multivariate model; a backward selection was performed to obtain the optimal reduced model. Results of the optimal multivariate model were presented as adjusted hazard ratio and 95% confidence intervals.

All statistical analyses were performed using SAS software v9.4 (Institute Inc., Cary, NC, USA, 25513). *p*-values < 0.05 were considered statistically significant. 

## 5. Conclusions

In this multicenter retrospective series of naTC patients who had pT3-4, pN+ disease or R1-2 resection, pEBRT improved LRF. We further observed that limited-field IMRT may be preferable. 

## Figures and Tables

**Table 1:** Patient and tumor characteristics and initial treatment for whole population and according to external beam radiotherapy.

	All Patients (*N* = 254)	Patients Without EBRT (*N* = 38)	Patients with EBRT (*N* = 216)	*p*-Value
Gender (Male)	117 (46.1%)	14 (36.8%)	103 (47.7%)	0.216
Age (years)				0.909
Median (min-max)	61 (51–69)	66 (57–70)	60 (49–69)	
≥ 45	206 (81.7%)	30 (81.1%)	176(81.9%)	
Performance status				0.273
0	138 (63.0%)	27 (73.0%)	111 (61.0%)	
1	72 (32.9%)	8 (21.6%)	64 (35.2%)	
2	9 (4.1%)	2 (5.4%)	7 (3.8%)	
Tumor size (cm)				0.312
Median (min-max)	4 (2–6)	3 (2–6)	4 (2–6)	
>4	77 (57.0%)	12 (48.0%)	65 (59.1%)	
T classification				0.089
Tx + T0	24 (10.7%)	1 (2.94%)	23 (12.2%)	
T1 + T2	63 (28.3%)	14 (41.2%)	49 (25.9%)	
T3 + T4	136 (61.0%)	19 (55.9%)	117 (61.9%)	
N classification				0.969
N0	89 (41.01%)	15 (42.9%)	74 (40.7%)	
N1	128 (59.0%)	20 (57.1%)	108 (59.4%)	
Metastatic disease	27 (11.02%)	5 (14.2%)	22 (10.5%)	0.558
UICC Stage				0.135
I + II + III	81 (38.57%)	17 (50%)	64 (36.4%)	
IV	129 (61.43%)	17 (50%)	112 (63.6%)	
Tracheal invasion	38 (16.9%)	3 (8.3%)	35 (18.6%)	0.132
Esophageal invasion	15 (6.8%)	2 (5.6%)	13 (7.1%)	1
Laryngeal invasion	6 (2.7%)	1 (2.8%)	5 (2.7%)	1
Histology (not exclusive)				
Papillary	171 (68.1%)	28 (73.7%)	143 (67.1%)	0.425
Follicular	22 (8.9%)	1 (2.7%)	21 (9.9%)	0.215
Poorly differentiated thyroid carcinoma	31 (12.5%)	5 (13.5%)	26 (12.3%)	0.790
Medullary	38 (15.3%)	5 (13.5%)	33 (15.6%)	0.749
Total thyroidectomy	217 (86.5%)	35 (92.1%)	182 (85.5%)	0.269
Lymph node dissection	209 (86.4%)	35 (92.1%)	174 (85.3%)	0.261
Quality of resection				0.027
R0	113 (51.4%)	24 (72.7%)	89 (47.6%)	
R1	67 (30.5%)	5 (15.1%)	62 (33.2%)	
R2	40 (18.2%)	4 (12.1%)	36 (19.2%)	
Extrathyroidal extension	160 (71.43%)	23 (67.6%)	137 (72.1%)	0.596
Microvascular invasion	37 (25.0%)	9 (31.0%)	28 (23.5%)	0.403
Perineural invasion	26 (18.8%)	10 (40.0%)	16 (14.2%)	0.008
Vascular embol	49 (32.9%)	15 (51.7%)	34 (28.3%)	0.016
Lymphatic embol	34 (22.8%)	11 (42.3%)	23 (18.7%)	0.009
Radioiodine fixation	158 (63.7%)	28 (73.7%)	130 (61.9%)	0.165
Radioiodine treatment	58 (24.0%)	3 (8.8%)	55 (26.4%)	0.026
Chemotherapy	35 (13.8%)	3(7.9%)	32 (14.8%)	0.254

Results presented as frequency and percentage unless otherwise indicated, Abbreviations: T: tumor, N: Nodal, min: minimum, max: maximum, R0: complete resection, R1: microscopic resection, R2: macroscopic resection, EBRT: External Beam Radiotherapy.

**Table 2:** Prognostic factors of locoregional recurrence after multiple imputations in univariate and multivariate analyses in the external beam radiotherapy (EBRT) group.

	Univariate Analyses		Multivariate Analysis *	
	HR and 95% CI	*p*	HR and 95% CI	*p*
Sex (Male vs. Female)	0.86 (0.48–1.55)	0.624		
Age (≥ 45 vs. <45)	1.33 (0.59;2.97)	0.492		
Performance status				
0	1			
1	1.07 (0.56;2.05)	0.832		
2	2.3 (0.7;7.53)	0.170		
Tumor size (≥4 vs. <4 cm)	1.12 (0.54;2.32)	0.756		
T classification				
Tx + T0	0.73 (0.24;2.26)	0.586		
T1 + T2	1			
T3 + T4	1.17 (0.58;2.33)	0.663		
N classification (N1 vs. N0)	1.14 (0.6;2.15)	0.693		
Metastasis disease	0.63 (0.20–2.02)	0.441		
Uicc stage (IV vs. I + II + III)	1.27 (0.68;2.4)	0.454		
Tracheal invasion	1.84 (0.92;3.66)	0.084		
Esophageal invasion	0.77 (0.19;3.11)	0.712		
Laryngeal invasion	3.76 (0.76;18.64)	0.105		
Histology				
Papillary	0.55 (0.31;1)	0.049		
Follicular	0.92 (0.33;2.54)	0.872		
PDTC	3.19 (1.61;6.35)	<0.001	2.56 (1.18;5.57)	0.018
Medullary	1.47 (0.69;3.12)	0.318		
Total thyroidectomy	0.37 (0.19;0.72)	0.003	0.48 (0.23;1.01)	0.051
Lymph node resection	0.94 (0.43;2.03)	0.865		
Quality of resection				
R0	1	1		
R1	1.1 (0.54;2.27)	0.786		
R2	1.94 (0.92;4.08)	0.082		
Extrathyroidal extension	1.14 (0.57;2.25)	0.716		
Perineural invasion	1.62 (0.74;3.55)	0.230		
Vascular embol	2.08 (1.02;4.23)	0.044		
Lymphatic embol	1.36 (0.6;3.11)	0.458		
Radioiodine fixation	0.81 (0.44;1.47)	0.479		
Radioiodine treatment	0.78 (0.37–1.62)	0.504		
Chemotherapy	1.60 (0.83–3.09)	0.162		
EBRT (for recurrence vs. for primary event)	1.46 (0.8;2.67)	0.213		
Target volume of EBRT (extensive vs. limited-field)	0.95 (0.42–2.19)	0.913		
Technique of EBRT (2D + 3D vs. IMRT, VMAT)	0.83 (0.45;1.51)	0.535		
Interruption of EBRT	2.41 (1.26;4.63)	0.008		

Abbreviations: Nb: Number, T: tumor, N: Nodal, M: Metastasis, EBRT: External Beam Radiotherapy, R0: complete resection, R1: microscopic resection, R2: macroscopic resection, 3D: 3-dimensional conformal radiotherapy, 2D: 2-dimensional radiotherapy, IMRT: Intensity modulated radiotherapy, VMAT: Volumetric modulated radiotherapy. * Optimal model after backward selection on parameters with a *p*-value less than 0.1 in bivariate analyses.

**Table 3:** Prognostic factors of overall survival after multiple imputations in univariate and multivariate analyses in the external beam radiotherapy (EBRT) group.

	Univariate Analysis		Multivariate Analysis *	
	HR and 95% CI	*p*	HR and 95% CI	*p*
Sex (Male vs. Female)	1.09 (0.69–1.73)	0.696		
Age (≥ 45 vs. <45)	3.1 (1.37;7.02)	0.007	3.5 (1.61;7.59)	0.001
Performance status				
0	1			
1	1.11 (0.65;1.9)	0.693		
2	1.61 (0.53;4.92)	0.403		
Tumor size (≥4 vs. <4 cm)	1.37 (0.79;2.37)	0.267		
T classification				
Tx + T0	1.35 (0.55;3.32)	0.509		
T1 + T2	1			
T3 + T4	2.63 (1.42;4.87)	0.002		
N classification (N1 vs. N0)	1.01 (0.62;1.64)	0.969		
Metastasis disease	1.31 (0.56-3.07)	0.534		
Uicc stage (IV vs. I + II + III)	1.79 (1.09;2.95)	0.022		
tracheal invasion	2.15 (1.21;3.84)	0.009		
Esophageal invasion	1.91 (0.65;5.61)	0.238		
Laryngeal invasion	5.87 (1.56;21.98)	0.009	6.66 (2.17;20.42)	<0.001
Histology				
Papillary	0.62 (0.39;0.99)	0.047		
Follicular	1.68 (0.91;3.08)	0.094		
PDTC	2.35 (1.29;4.28)	0.005		
Medullary	1 (0.53;1.9)	0.990		
Total thyroidectomy	0.41 (0.24;0.71)	0.001		
Lymph node resection	0.97 (0.52;1.8)	0.918		
Quality of resection				
R0	1			
R1	1.4 (0.81;2.44)	0.231		
R2	2.5 (1.35;4.62)	0.004		
Extrathyroidal extension	1.74 (0.98;3.12)	0.061		
Perineural invasion	1.03 (0.47;2.29)	0.935		
Vascular embol	1.76 (0.98;3.17)	0.059		
Lymphatic embol	1.92 (0.98;3.76)	0.057		
Radioiodine fixation	0.6 (0.37;0.95)	0.029		
Radioiodine treatment	0.53 (0.29;0.96)	0.036	0.41 (0.22;0.74)	0.003
Chemotherapy	1.49 (0.91-2.47)	0.113		
EBRT (for recurrence vs. for primary event)	0.93 (0.58;1.48)	0.747		
Target volume of EBRT (extensive vs. limited-field)	0.94 (0.47-1.86)	0.854		
Technique of EBRT (2D+3D vs. IMRT, VMAT)	2.03 (1.14;3.6)	0.016	2.42 (1.33;4.39)	0.004
Interruption of EBRT	2.87 (1.77;4.66)	<0.001	2.82 (1.58;5.04)	<0.001

Abbreviations: Nb: Number, T: tumor, N: Nodal, M: Metastasis, EBRT: External Beam Radiotherapy, R0: complete resection, R1: microscopic resection, R2: macroscopic resection, 3D: 3-dimensional conformal radiotherapy, 2D: 2-dimensional radiotherapy, IMRT: Intensity modulated radiotherapy, VMAT: Volumetric modulated radiotherapy. * Optimal model after backward selection on parameters with a *p*-value less than 0.1 in bivariate analyses.

## Supplementary Materials

The following are available online at https://www.mdpi.com/2072-6694/11/6/849/s1, Table S1: Description of the number of missing data in the 254 analyzed patients; Table S2: Prognostic factors of locoregional recurrence without multiple imputations in the EBRT group: complete-case analysis; Table S3: Prognostic factors of overall survival without multiple imputations in the EBRT group: complete-case analysis.
