# Supplementary material for: 10-Year Locoregional Control with Postoperative External Beam Radiotherapy in Patients with Locally Advanced High-Risk Non-Anaplastic Thyroid Carcinoma De Novo or at Relapse, a Propensity Score Analysis
Source: Cancers (Basel). 2019 Jun 19;11(6):849. doi: 10.3390/cancers11060849 (PMC6628348; doi:10.3390/cancers11060849)
Supplement: Supplementary file 1 [file cancers-11-00849-s001.pdf]

Table S1: Description of the number of missing data in the 254 analyzed patients.

**Age**	**2(0.8%)**
Physical status	35(13.8%)
Tumor size	119(46.9%)
T classification	31(12.2%)
N classification	37(14.6%)
M1	9(3.5%)
UICC Stage	44(17.3%)
Tracheal invasion	30(11.8%)
Esophageal invasion	35(13.8%)
Laryngeal invasion	34(13.4%)
Histology	19(7.5%)
Total thyroidectomy	3(1.2%)
Lymph node dissection	12(4.7%)
Quality of resection	34(13.4%)
extrathyroidal extension	30(11.8%)
microvascular invasion	106(41.7%)
perineural invasion	116(45.7%)
vascular embol	105(41.3%)
lymphatic embol	105(43.3%)
Radioiodine fixation	6(3.4%)
Radioiodine treatment	12(4.8%)
Indication of treatment*	1(0.4%)
Target volume*	3(1.2%)
Technique of EBRT*	15(9.9%)
Total dose *	39(15.3%)
Interruption of EBRT*	12(4.8%)

Results presented as frequency and percentage. Abbreviations: Nb: Number, T: tumor, N: Nodal, M: Metastasis, EBRT: External Beam Radiotherapy. *Only in the EBRT group.

Table S2: Prognostic factors of locoregional recurrence without multiple imputations in the EBRT group : Complete-case analysis.

	**Univariate Analyses**		**Multivariate Analysis***	
	HR and 95% CI	p	HR and 95% CI	*p*
Sex (Male vs Female)	1.1(0.69;1.74)	0.697		
Age (≥ 45 vs <45)	3.15(1.36;7.29)	0.007		
Performance status				
0	1			
1	1.22(0.71;2.1)	0.474		
2	1.78(0.63;5.05)	0.275		
Tumor size (≥4 vs <4 cm)	1.62(0.88;3.02)	0.124		
T classification				
Tx + T0	1.52(0.60;3.87)	0.382	1.93(0.74;5.04)	0.180
T1 + T2	1		1	
T3 + T4	3.00(1.50;5.98)	0.002	2.84(1.40;5.74)	0.004
N classification (N1 vs N0)	1.15(0.68;1.94)	0.599		
Metastasis disease	1.31(0.56;3.06)	0.531		
Uicc stage (IV vs I + II + III)	1.97(1.12;3.47)	0.018		
Tracheal invasion	2.34(1.34;4.08)	0.003		
Esophageal invasion	1.90(0.76;4.76)	0.170		
Laryngeal invasion	6.09(1.85;20.04)	0.003	8.23(2.38;28.46)	<0.001
Histology				
Papillary	0.62(0.39;1.01)	0.053		
Follicular	1.61(0.8;3.26)	0.183		
PDTC	2.35(1.28;4.31)	0.006		
Medullarry	1.02(0.54;1.94)	0.953		
Total thyroidectomy	0.41(0.24;0.71)	0.001		
Lymph node resection	1.05(0.53;2.06)	0.883		
Quality of resection				
R0	1			
R1	1.59(0.87;2.89)	0.132		
R2	2.81(1.47;5.38)	0.002		
Extrathyroidal extension	1.96(1.04;3.69)	0.037		
Microvascular invasion	1.79(0.92;3.51)	0.088		
Perineural invasion	0.95(0.4;2.22)	0.897		
Vascular embol	2.04(1.11;3.78)	0.023		
Lymphatic embol	1.75(0.86;3.54)	0.120		
Radioiodine fixation	0.60(0.37;0.96)	0.032		
Radioiodine treatment	0.52(0.28;0.98)	0.042		
Chemotherapy	1.50(0.83;2.70)	0.177		
Treatment of EBRT (for recurrence vs for primary event)	0.91(0.57;1.45)	0.694		
Target volume of EBRT (extensive vs limited-field)	0.94(0.48;1.84)	0.851		
Technique of EBRT (2D + 3D vs IMRT,VMAT)	2.00(1.13;3.56)	0.018		
Interruption of EBRT	2.81(1.57;5.02)	<0.001	3.70(1.95;7.03)	<0.001

Abbreviations: Nb: Number, T: tumor, N: Nodal, M: Metastasis, EBRT: External Beam Radiotherapy, R0: complete resection, R1: microscopic resection, R2: macroscopic resection, 3D: 3-dimensional conformal radiotherapy, 2D: 2-dimensional radiotherapy, IMRT: Intensity modulated radiotherapy, PDTC: poor-differentiated thyroid carcinoma, VMAT: Volumetric modulated radiotherapy. *Optimal model after backward selection on parameters with a *p*-value less than 0.1 in bivariate analyses.

Table S3: Prognostic factors of overall survival without multiple imputations in the EBRT group: complete-case analysis.

	Univariate Analyses		Multivariate Analysis*	
	HR and 95% CI	*p*	HR and 95% CI	*p*
Sex (Male vs Female)	1.1(0.69;1.74)	0.697		
Age (≥ 45 vs <45)	3.15(1.36;7.29)	0.007		
Performance status				
0	1			
1	1.22(0.71;2.1)	0.474		
2	1.78(0.63;5.05)	0.275		
Tumor size (≥4 vs <4 cm)	1.62(0.88;3.02)	0.124		
T classification				
Tx + T0	1.52(0.60;3.87)	0.382	1.93(0.74;5.04)	0.180
T1 + T2	1		1	
T3 + T4	3.00(1.50;5.98)	0.002	2.84(1.40;5.74)	0.004
N classification (N1 vs N0)	1.15(0.68;1.94)	0.599		
Metastasis disease	1.31(0.56;3.06)	0.531		
Uicc stage (IV vs I + II + III)	1.97(1.12;3.47)	0.018		
Tracheal invasion	2.34(1.34;4.08)	0.003		
Esophageal invasion	1.90(0.76;4.76)	0.170		
Laryngeal invasion	6.09(1.85;20.04)	0.003	8.23(2.38;28.46)	<0.001
Histology				
Papillary	0.62(0.39;1.01)	0.053		
Follicular	1.61(0.8;3.26)	0.183		
PDTC	2.35(1.28;4.31)	0.006		
Medullarry	1.02(0.54;1.94)	0.953		
Total thyroidectomy	0.41(0.24;0.71)	0.001		
Lymph node resection	1.05(0.53;2.06)	0.883		
Quality of resection				
R0	1			
R1	1.59(0.87;2.89)	0.132		
R2	2.81(1.47;5.38)	0.002		
Extrathyroidal extension	1.96(1.04;3.69)	0.037		
Microvascular invasion	1.79(0.92;3.51)	0.088		
Perineural invasion	0.95(0.4;2.22)	0.897		
Vascular embol	2.04(1.11;3.78)	0.023		
Lymphatic embol	1.75(0.86;3.54)	0.120		
Radioiodine fixation	0.60(0.37;0.96)	0.032		
Radioiodine treatment	0.52(0.28;0.98)	0.042		
Chemotherapy	1.50(0.83;2.70)	0.177		
Treatment of EBRT (for recurrence vs for primary event)	0.91(0.57;1.45)	0.694		
Target volume of EBRT (extensive vs limited-field)	0.94(0.48;1.84)	0.851		
Technique of EBRT (2D + 3D vs IMRT,VMAT)	2.00(1.13;3.56)	0.018		
Interruption of EBRT	2.81(1.57;5.02)	<0.001	3.70(1.95;7.03)	<0.001

Abbreviations: Nb: Number, T: tumor, N: Nodal, M: Metastasis, EBRT: External Beam Radiotherapy, R0: complete resection, R1: microscopic resection, R2: macroscopic resection, 3D: 3-dimensional conformal radiotherapy, 2D: 2-dimensional radiotherapy, IRMT: Intensity modulated radiotherapy, VMAT: Volumetric modulated radiotherapy, *Optimal model after backward selection on parameters with a *p*-value less than 0.1 in bivariate analyses.
